# A Case of Risperidone-Induced Urinary Incontinence

**DOI:** 10.7759/cureus.58590

**Published:** 2024-04-19

**Authors:** Allison C Eierman, Alexandra E Reischman, Robert V Gouthro

**Affiliations:** 1 Psychiatry, Medical College of Wisconsin, Green Bay, USA; 2 Psychiatry, Medical College of Wisconsin, Wausau, USA

**Keywords:** medication compliance, schizoaffective disorder, atypical antipsychotic, male urinary incontinence, risperidone adverse effect

## Abstract

Schizophrenia spectrum disorders are psychiatric conditions associated with an increased risk of all-cause mortality; patients with these conditions have a shortened average lifespan compared to the general population. First-line treatment for schizophrenia spectrum illness consists of atypical antipsychotics, which are associated with well-understood side effects, including metabolic syndrome, anticholinergic effects, and extrapyramidal symptoms. We are presenting a case of a 36-year-old patient treated with the atypical antipsychotic risperidone who experienced treatment-associated urinary incontinence. In the current literature, atypical antipsychotic-induced urinary incontinence is not well-documented in patients with schizophrenia spectrum disorder. Incontinence is often a topic of societal shame for many patients, and as a side effect, it may influence medication compliance. In the treatment of schizophrenia spectrum disorders, compliance is essential to prevent psychosis relapse in patients, so prescribers must be aware of this potential side effect and how to manage it. Upon a patient presenting with incontinence suspected to be due to atypical antipsychotics, other more common causes of incontinence must first be ruled out. Then, further management can consist of stopping the offending medication or adding a medication to address the incontinence. In this case, our patient had an extended history of suboptimal treated schizoaffective disorder, and risperidone was providing significant improvement; therefore, to ensure continued improvement, we initiated oxybutynin to manage urinary incontinence.

## Introduction

The lifetime prevalence of all psychotic disorders, including schizophrenia spectrum illness, is approximately 3% [1]. Schizophrenia spectrum illnesses, including schizophrenia and schizoaffective disorder, are psychiatric conditions involving chronic or reoccurring positive symptoms of psychosis (hallucinations, delusions, etc.) as well as negative symptoms such as avolition and anhedonia. People with schizophrenia and schizoaffective disorder have an increased all-cause mortality and die approximately 15-20 years prematurely compared to the general population [2,3]. Treatment for schizophrenia spectrum illness consists primarily of pharmacotherapy with antipsychotic medications. Unfortunately, medication non-adherence ranges between 63-74% in patients with schizophrenia and increases the risk for symptom relapse by nearly 5 times [4,5]. A meta-analysis evaluating risk factors of medication noncompliance reported that drug factors, such as poor drug efficacy or high medication side effect response, had the greatest impact on adherence. Furthermore, the severity of the side effects of drugs will lead to patient non-compliant behavior [6]. Therefore, physicians prescribing antipsychotic medications to patients must be aware of potential side effects, their perceived severity in society, and how to address these challenges to ensure patient compliance. 

Second-generation antipsychotics, also known as atypical antipsychotics, are a first-line medical treatment for schizophrenia spectrum disorders. These medications act as antagonists of dopamine, serotonin, alpha-adrenergic, histamine, and muscarinic receptors with varying degrees of affinity [7]. Common side effects associated with atypical antipsychotics include weight gain, metabolic syndrome, sedation, dry mouth, dry eyes, extrapyramidal symptoms, hyperprolactinemia, cardiovascular complications such as QT prolongation and cardiomyopathies, cataracts, and sexual dysfunction. Urinary incontinence is a less understood complication of second-generation antipsychotic drugs, and the incidence of this adverse effect has not been well established. Urinary incontinence and/or nocturnal enuresis may occur in only 0.2% of patients, but ranges up to 42% have been found in the literature [8-11], making the actual incidence of antipsychotic-induced urinary incontinence unclear. Currently, in the United States, the research on urinary incontinence as a side effect of atypical antipsychotic use for schizophrenic spectrum disorders is limited to a small number of case studies. 

Urinary incontinence is a prevalent condition experienced in a variety of patient populations; however, it carries a stigma that leads to a discrepancy between those experiencing incontinence and those seeking diagnosis or management [12]. An epidemiological systematic review found that most studies estimate the prevalence of urinary incontinence between 25-45% in women and between 11-34% in men [13]. However, some studies suggest that only approximately 1/3rd of patients experiencing incontinence will seek help from a medical provider [14]. This discrepancy may be attributed to the taboo nature that incontinence carries and how it is perceived by society. The perception of urinary incontinence varies between patient populations, which may exacerbate this discrepancy [15]. Due to society's shameful and embarrassing perception of incontinence, likely, incontinence as a medication side effect may negatively impact patient compliance. Medication compliance is crucial for patients with Schizophrenia in preventing psychosis relapse, so it is essential to be aware of incontinence as a potential adverse effect of atypical antipsychotics and how to address it to ensure patient safety. 

## Case presentation

A 36-year-old male was referred to a free mental health clinic (FMHC) by primary care for psychiatric evaluation. Referral history included reports of an "underlying mental health condition" with known prior use of escitalopram, olanzapine, fluoxetine, mirtazapine, and temazepam. The primary care visit documentation also relayed the patient reported hearing voices that "scream at him to harm others," difficulty sleeping, restlessness, and racing thoughts. 

Upon presentation to the FMHC, the patient denied knowledge that the visit was focused on his mental health. He reported having "anxiety, just like everyone does." He denied the need for medications, declined to answer mental health-related questions, and, after only a few minutes, abruptly left the exam room and clinic. 

Later that morning, the patient's family presented to the clinic to report significant concerns with the patient's mental health state. They reported frustration with years of medication ineffectiveness and noncompliance in both the United States and Mexico. They reported that the patient had a long history of psychosis and depressive symptoms. Immediate concerns included internal preoccupation, auditory hallucinations, persecutory delusions, aggressive behaviors, and a recurring fear he may harm himself or others. His actions had led to disruptions in the neighborhood and police involvement. All recent attempts to provide mental health assistance have failed. 

The FMHC provided education to the family, including the legal grounds for evaluations, emergency detentions, third-party petitions, and local support resources. The family was encouraged to ask the patient to return for another visit. 

Approximately 10 months later, the patient and his family reported to the clinic for evaluation. The patient reported hearing voices that tell him "good and bad things," but he denied visual hallucinations. The patient reported feeling sad and conveyed a want "to get better and feel happy more". His family reiterated many of the same concerns previously shared. Based on the historical report and patient presentation, the patient was diagnosed with schizoaffective disorder and depressive type and started on risperidone 2mg daily. A follow-up was scheduled. 

Upon follow-up, the patient reported medication adherence, improved mood, and decreased "voices." The family relayed an improvement in patient interactions and decreased safety concerns. They felt he seemed more relaxed and participated more fully in self-care. The family requested continued titration of the risperidone, and the patient agreed. When inquiring about side effects, it was mentioned the patient had increased urinary frequency and incontinence. Although noted, the family believed his improvement was significant and denied interest in alternative options. His risperidone dosage was titrated to 2 mg twice daily, and they were asked to call the clinic if urinary incontinence continued. 

His urinary symptoms worsened, with the patient reporting increased nocturnal enuresis, occasional voids in his clothing during the day, and skin irritation due to exposure to wet clothing. The patient denied dysuria, hematuria, and penile discharge. With assistance from his primary care provider, underlying medical causes, including gonorrhea, chlamydia, acute cystitis, prostate enlargement, and diabetes, were ruled out. The primary care provider consulted the FMHC, and the pros and cons of transitioning to an alternative medication were discussed. Given that the patient and family were happy with the improvements seen at this increased dosage and therefore wary of a change, an oxybutynin 24-hour formulation was initiated to address his incontinence concern. 

The following month, the patient presented for follow-up and reported continued mental health improvement. He reported no longer feeling depressed and denied continued auditory hallucinations. His family felt the patient was functioning at a baseline not seen in many years, and risperidone was believed to be more effective than past psychotropics. Oxybutynin initiation reportedly led to the full resolution of his urinary symptoms. 

## Discussion

This case is shared to highlight an often overlooked but significant adverse effect of risperidone. Although sporadic cases of urinary incontinence secondary to antipsychotic use can be found in the literature, thus far, the previously documented cases have occurred exclusively in children, often children with autism or other developmental disorders, and few documented cases originate in the United States. To our knowledge, this is the first documented case of an adult male with new-onset urinary incontinence following the initiation of risperidone.

Although not life-threatening, urinary incontinence may have a profound impact on both patients and their caregivers. Incontinence episodes may be a source of embarrassment and shame. Fear of incontinence episodes may lead patients to limit their social activities in an effort to minimize the risk of incontinence outside of the home. Such isolation further contributes to poor self-confidence and mental health outcomes. For patients who rely on help from family or caregivers, incontinence episodes may make maintaining hygiene difficult, and the associated increase in patient care may contribute to caregiver burnout. Most significantly, urinary incontinence and the sequelae described above may lead to medication non-adherence and subsequent return of psychotic symptoms.

Despite its significance, few studies have examined urinary incontinence secondary to antipsychotic use, and the mechanism behind this adverse effect remains unclear. A 2009 review of urodynamic patterns of 15 patients with schizophrenia taking second-generation antipsychotics showed detrusor overactivity in 33.3% of patients as well as reduced bladder compliance in 41.6% of their sample [16]. However, the same study found no evidence of abnormal urodynamic patterns in 25% of their sample. Given the small sample size and mixed results, this suggests that further research is needed to understand which patients may be most at risk for urinary incontinence. If detrusor overactivity is the cause of incontinence, this may explain why our patient was able to be treated successfully with oxybutynin, an anticholinergic medication that relaxes the detrusor muscle.

Another possible pathway for antipsychotic-induced urinary incontinence involves urethral sphincter tone. Although risperidone primarily acts as a serotonin (5HT2a) and dopamine (D2) receptor antagonist, this medication also exhibits strong antagonism of alpha-adrenergic receptors (a1 and a2). The alpha-1 adrenergic system regulates the tone of the internal urethral sphincter. Therefore, atypical antipsychotics with a larger affinity for alpha receptors may be at increased risk of urinary incontinence due to lack of urethral sphincter tonus. Based on this mechanism, it would be reasonable for providers to discontinue the offending medication and initiate a different atypical antipsychotic, such as sulpiride, amisulpiride, or aripiprazole, which have a lower affinity for alpha-1 receptors [17]. Unfortunately, sulpiride and amisulpride are not available in the US.

When new urinary incontinence is identified in a patient, medical explanations must be thoroughly evaluated before a medication management change. We recommend that patients be evaluated for common causes of urinary incontinence, including gonorrhea, chlamydia, acute cystitis, prostate enlargement, and diabetes, as demonstrated in the clinical history above. Medication interactions should also be considered, including over-the-counter medication and supplement use. Once medical causes have been ruled out, providers have several management options. Providers may wish to reduce the risperidone (or other) antipsychotic dose to one that does not produce urinary incontinence or minimizes episodes to a frequency that is not distressing to the patient. If the dose is reduced, care must be taken to ensure that mental health symptoms are still controlled at the reduced dose. Alternatively, the patient may be transitioned to a different antipsychotic, although this strategy's efficacy is unclear. If the patient is otherwise stable on an antipsychotic, the risks of transiting (decompensation of mental health, risk of additional side effects, etc.) must be carefully considered by both patient and provider.

Providers may consider medication management for patients unable to tolerate a decrease in dose or switch medications. As demonstrated in our patient's case, oxybutynin or a similar anticholinergic medication may be a reasonable treatment option. Case studies have also shown pseudoephedrine, an alpha-adrenergic agonist, to be effective in treating clozapine-induced urinary incontinence, and this medication may be effective in treating incontinence secondary to risperidone and other antipsychotics [18]. Mirabegron, botox injections, imipramine, and duloxetine are other treatment options commonly used for urinary incontinence [19]. To our knowledge, there have been no documented cases describing the use of these treatments in antipsychotic-induced incontinence, but providers should keep an open mind to these medications, especially if other management options have failed or for patients with co-occurring mood/anxiety disorders.

Regardless of the primary management strategy, providers are also advised to educate patients on behavioral modifications to reduce the frequency of incontinence episodes. Most common behavioral methods include bladder training, biofeedback, and pelvis muscle exercises to strengthen key muscles involved in sphincter contraction. Timed and/or prompted voiding may also be helpful, especially for children or those with intellectual disabilities. Lifestyle modifications, including fluid management, caffeine reduction, avoidance of potential bladder irritants (sugar substitutes, citrus, spicy foods, and tomato products), and weight management, may provide additional relief of symptoms [20].

## Conclusions

Urinary incontinence is a poorly understood but potentially significant side effect of risperidone and other second-generation antipsychotics. If left untreated, urinary incontinence can negatively impact quality of life and contribute to medication non-adherence and decompensation. Although several management options are discussed, due to the scarcity of research, it is unclear if one management strategy is superior to another or how to predict which patient may be most at risk. We believe increased awareness of this side effect is the first step towards identifying affected patients, understanding the consequences of untreated urinary incontinence, exploring treatment options, and improving patient outcomes.
